# Supplementary material for: Interactions between stimulus and response types are more strongly represented in the entorhinal cortex than in its upstream regions in rats
Source: eLife. 2017 Dec 27;6:e32657. doi: 10.7554/eLife.32657 (PMC5771666; doi:10.7554/eLife.32657)
Supplement: Transparent reporting form [file elife-32657-transrepform.docx]

***eLife’s* transparent reporting form**

We encourage authors to provide detailed information *within their submission* to facilitate the interpretation and replication of experiments. If you have any questions, please contact us: [editorial@elifesciences.org](mailto:editorial@elifesciences.org).

**Sample-size estimation**

- You should state whether an appropriate sample size was computed when the study was being designed
- You should state the statistical method of sample size computation and any required assumptions
- If no explicit power analysis was used, you should describe how you decided what sample (replicate) size (number) to use

Please outline where this information can be found within the submission (e.g., page numbers or figure legends), or explain why this information doesn’t apply to your submission:

1. In this study we used repeated-measures ANOVAs to analyze drug effects between the ACSF and MUS groups. Power analysis did not help very much to determine the adequate sample size in our study because we could obtain the actual power of 1 with the sample size of 2 based on a power analysis (using G*Power3.1 by Faul et al., 2007) with the following parameters (see below):

   SCN-NSR task: effect size f=11.363; alpha error probability=0.05; power=0.95; *number of group=1*; number of measurement=3; correlation among representative measures=0.4123; non-sphericity correction=1

SCN-SR task: effect size f=12.745; alpha error probability=0.05; power=0.95; *number of group=1*; number of measurement=3; correlation among representative measures=0.119; non-sphericity correction=1

OBJ-NSR task: effect size f=15.548; alpha error probability=0.05; power=0.95; *number of group=1*; number of measurement=3; correlation among representative measures=0.3766; non-sphericity correction=1

OBJ-SR task: effect size f=7.611; alpha error probability=0.05; power=0.95; *number of group=1*; number of measurement=3; correlation among representative measures=0.2126; non-sphericity correction=1

vOBJ-NSR task: effect size f=40.477; alpha error probability=0.05; power=0.95; *number of group=1*; number of measurement=3; correlation among representative measures=0.3156; non-sphericity correction=1

Therefore, the sample size for each experiment was determined by considering both our prior experience and the field standard. Specifically, in our study, six to eight rats were pre-trained across many days and tested with drug injections using a within-subject design. As the power analysis shows, the number of rat is sufficient to conclude the results of our behavioral studies. Furthermore, we have repeatedly shown through our previous studies (see references) that the number of subjects used in the current study is adequate to draw a firm conclusion. This is usually the case when an animal is first trained up to criterion and tested using a within-subject drug injection schedule, compared to one shot test using between-group comparisons (e.g., fear conditioning).

References:

1. Jo, Y.S. and Lee, I. (2010) Journal of Neuroscience 30:9850-9858.
2. Kim, J. and Lee, I. (2011) Hippocampus 21:609-621.
3. Kim, S. et al. (2012) Front Behav Neurosci 6:66.
4. Ahn, J.R. and Lee, I. (2015) Journal of Neuroscience 35(4):1692-1705.

Information for sample size was given as follows:

1. Page 4: repeated-measures ANOVA to test the drug effect in the SCN-NSR task (n=7)
2. Page 4: repeated-measures ANOVA to test the drug effect in the SCN-SR memory task (n=8)
3. Page 5: repeated-measures ANOVA to test the drug effect in the OBJ-NSR memory task (n=7)
4. Page 5: repeated-measures ANOVA to test the drug effect in the OBJ-SR memory task (n=6)
5. Page 6: repeated-measures ANOVA to test the interaction effect between region and task in the NSR tasks. (n=7)
6. Page 6: repeated-measures ANOVA to test the interaction effect between region and task in the SR tasks. (n=14)

**Replicates**

- You should report how often each experiment was performed
- You should include a definition of biological versus technical replication
- The data obtained should be provided and sufficient information should be provided to indicate the number of independent biological and/or technical replicates
- If you encountered any outliers, you should describe how these were handled
- Criteria for exclusion/inclusion of data should be clearly stated
- High-throughput sequence data should be uploaded before submission, with a private link for reviewers provided (these are available from both GEO and ArrayExpress)

Please outline where this information can be found within the submission (e.g., page numbers or figure legends), or explain why this information doesn’t apply to your submission:

As shown in our raw data, we replicated the behavioral effects of muscimol inactivation in the PER and POR across subjects across different testing time points:

SCN-NSR task: 7 different rats experimented from mid 2015 until early 2016.

SCN-SR task: 8 different rats experimented from mid 2015 until mid 2016.

OBJ-NSR task: 7 different rats experimented from early 2015 until late 2015.

OBJ-SR task: 6 different rats experimented from early 2017 until mid 2017.

The replication of similar results for the drug injections at different time periods across different rats strongly suggests that the major drug effects reported in the current study are not due to other confounding or uncontrolled factors.

**Statistical reporting**

- Statistical analysis methods should be described and justified
- Raw data should be presented in figures whenever informative to do so (typically when N per group is less than 10)
- For each experiment, you should identify the statistical tests used, exact values of N, definitions of center, methods of multiple test correction, and dispersion and precision measures (e.g., mean, median, SD, SEM, confidence intervals; and, for the major substantive results, a measure of effect size (e.g., Pearson's r, Cohen's d)
- Report exact p-values wherever possible alongside the summary statistics and 95% confidence intervals. These should be reported for all key questions and not only when the p-value is less than 0.05.

Please outline where this information can be found within the submission (e.g., page numbers or figure legends), or explain why this information doesn’t apply to your submission:

(For large datasets, or papers with a very large number of statistical tests, you may upload a single table file with tests, Ns, etc., with reference to page numbers in the manuscript.)

Drug effects of the major four tasks were analyzed by using repeated-measures ANOVAs because ACSF and MUS injections into the PER or POR in the same rat. Specific values and statistics are provided in the manuscript as follows:

1. Repeated-measures ANOVA to test the main effect of drug in the SCN-NSR task (p.4)
2. Repeated-measures ANOVA to test the main effect of drug in the SCN-SR task (p.4)
3. Repeated-measures ANOVA to test the main effect of drug in the OBJ-NSR task (p.5)
4. Repeated-measures ANOVA to test the main effect of drug in the OBJ-SR task (p.5)

**Additional data files (“source data”)**

- We encourage you to upload relevant additional data files, such as numerical data that are represented as a graph in a figure, or as a summary table
- Where provided, these should be in the most useful format, and they can be uploaded as “Source data” files linked to a main figure or table
- Include model definition files including the full list of parameters used
- Include code used for data analysis (e.g., R, MatLab)
- Avoid stating that data files are “available upon request”

Please indicate the figures or tables for which source data files have been provided:

We also included raw performance data used for graphs in the manuscript as source data tables.
